# Association of Sports Participation and Diet with Motor Competence in Austrian Middle School Students

**DOI:** 10.3390/nu10121837

**Published:** 2018-11-29

**Authors:** Clemens Drenowatz, Klaus Greier

**Affiliations:** 1 Division of Physical Education, University of Education Upper Austria, 4020 Linz, Austria; 2 Division of Physical Education, Private University of Education (KPH-ES), 6422 Stams, Austria; nikolaus.greier@kph-es.at; 3 Department of Sport Science, University of Innsbruck, 6020 Innsbruck, Austria

**Keywords:** physical activity, food intake, adolescents, movement skills, fitness

## Abstract

Physical activity and diet are important contributors to overall health and development in adolescents. There remains, however, limited research on the combined association of sports participation and dietary pattern on motor competence, which is crucial for an active lifestyle during and beyond adolescence. The present study, therefore, examined the association between sports participation, dietary pattern, and motor competence in 165 middle school students (55% male) between 11 and 14 years of age. Body weight and height were measured, and motor competence was determined via the German motor test during regular Physical Education (PE). Further, participants completed a food frequency questionnaire and reported their engagement in club sports. Of the total sample 20% were overweight/obese and 49% reported participation in club sports, with no differences between boys and girls. Interaction effects of sports participation and dietary pattern on motor competence were limited, but sports participation and healthy diet were independently associated with higher motor performance. Healthy dietary choices, along with participation in club sports, therefore, should be promoted in adolescents in order to facilitate motor development. As adolescence is a crucial time for the establishment of lifelong behaviors, such efforts could facilitate a healthy lifestyle throughout adulthood.

## 1. Introduction

Low levels of physical activity (PA) and poor dietary choices are considered key health risk factors in youth [1]. In fact, 70% of premature deaths are attributed to behavioral choices begun during adolescent years [2]. Accordingly, adolescence provides a crucial window of opportunity for sustainable health promotion, including sufficient PA and healthy dietary choices [3]. Current guidelines emphasize a minimum of 60 min of moderate-to-vigorous PA [4], along with a diverse dietary intake that includes high consumption of leafy greens, fruits and vegetables, poultry, fish, and dairy while the consumption of fat and sugars should be limited [5]. Nevertheless, sedentary choices during leisure time, along with prolonged sitting times during school time are common in youth [6,7,8], and many children and adolescents do not meet current dietary recommendations [9,10]. Low PA levels most likely also contributed to a decline in physical fitness and motor competence in children and adolescents [11,12], which is a crucial contributor to a sustainable active lifestyle, as well as overall health and well-being [13,14].

In light of these trends, organized PA, including club sports, provides important opportunities for PA and the establishment of healthy behaviors [15,16]. In fact, a study in Australian adolescents between 10 and 16 years of age showed that they accrued the majority of their total PA during organized PA such as club sports [17]. Participation in sports during childhood and adolescence has also been associated with higher PA levels during adulthood [18,19]. With more than two-thirds of children and adolescents in various European countries participating in club sports, this may also be a viable setting for interventions targeting an active and healthy lifestyle [20]. In addition to beneficial associations with overall PA [21,22,23], participation in club sports has been associated with increased physical fitness and motor performance [24,25], as well as beneficial socio-emotional outcomes [26] and higher academic achievement [27,28]. A recent study further indicates beneficial associations between club sports participation and food intake [29], but the overall evidence on this relationship remains equivocal [30]. This may, at least partially, be attributed to higher energy needs in more active youth [31]. Accordingly, there appears to be a positive association between physical fitness and energy intake in children and adolescents [31,32]. Nevertheless, higher physical fitness has been associated with healthier dietary choices in adolescents, while this association is less clear in children [33,34,35].

Limited research, however, is available on the combined association of dietary pattern and sports participation with motor competence. Given the complex interaction between behavioral choices (i.e., diet and sports) and motor development, and the importance of motor competence in the promotion of an active lifestyle [36], such information may help with the refinement of current interventions, as well as the development of new strategies for the promotion of motor development in youth. The present study, therefore, examines the combined and independent association of club sports participation and dietary habits with motor competence in Austrian middle-school students. It was hypothesized that club sports participation, as well as healthy dietary patterns, are positively associated with motor competence. Further, it was hypothesized that the association between dietary pattern and motor competence is more pronounced in adolescents not participating in club sports.

## 2. Materials and Methods

A convenience sample of nine classes between grades 6 and 8 from middle schools in the Federal State of Tyrol, Austria, were selected for participation, resulting in 172 eligible participants between 11 and 14 years of age. Parents were informed about the nature of the study via mail, and provided written informed consent. Oral assent was obtained from the participants at the time of data collection, which occurred during May and June of 2018. The study was performed according to the ethical standards of the 2008 Declaration of Helsinki, with the study protocol being approved by the Institutional Review Board of the University of Innsbruck, the school board of the Federal State of Tyrol and the principals of the participating schools (approval number: 16/2017).

Anthropometric measurements. Body weight (kg) and height (cm) were measured according to standard procedures during a physical education class, by trained technicians, with participants wearing gym clothes and being barefoot. Body weight was measured to the nearest 0.1 kg with a gauged body scale (SECA^®^ 803, Seca, Hamburg, Germany), and height was measured to the nearest 0.1 cm with a mobile stadiometer (SECA^®^ 217, Seca, Hamburg, Germany). Body mass index (BMI, kg/m^2^) was calculated and converted to BMI percentiles based on the German reference system [37]. Subsequently, participants were classified as non-overweight/obese or overweight/obese using the 90th percentile.

Motor Competence. Following anthropometric measurements, motor competence was assessed via the German motor test (Deutscher Motorik Test, DMT6-18), which consists of eight test items to evaluate endurance, power, speed, coordination, and agility [38]. Specifically, the DMT6-18 consists of a 20 m sprint, sideways jumping, standing long jump, sit ups, push-ups, backwards balancing, a stand and reach test, and a 6 min run. After a standardized 5 min warm-up, participants started the test with the 20 m sprint. Other tests were completed in random order, except for the 6 min run, which was completed at the end of the session. All tests, including practice trials, were administered in accordance with the specifications provided by the test manual [38]. In addition to raw performance scores, age- and sex-specific standardized values were calculated based on a German reference sample, which were used in the statistical analyses. The mean of these standardized scores was further used to calculate an overall motor competence score.

Dietary assessment. Dietary information was obtained via a standardized food frequency questionnaire that has been used previously with Austrian adolescents [39]. The questionnaire was administered by a trained technician during regular class time. Participants reported the frequency (days/week) of the consumption of 42 foods that were subsequently summarized into food groups. Principal component analysis was used to identify dietary patterns. The analysis revealed three factors with an Eigenvalue >1, which explained 55.9% of the total variance of dietary intake. Specifically, factor 1 was characterized by high loadings of meat, fish, bread (white and/or wholemeal), pasta and sweets consumption (meat/carbohydrates (CHO)); factor 2 was characterized by high loadings of milk, cereal, nuts and fruits (milk/cereal); and factor 3 was characterized by high consumption of water and vegetables, as well as low consumption of fast food (FF) and soft drinks (water/low FF).

Club sports participation. Participants also reported whether they participated in club sports, and how much time was spent in organized PA. Due to the limited variability in time spent in club sports, participants were stratified into club sports or no club sports for subsequent analyses.

Statistical Analysis. Descriptive statistics were calculated, and data was checked for normal distribution. Sex differences for club sports participation and weight status were determined via Chi-square tests, while differences in motor competence and dietary pattern were examined via multivariate analysis of variance (MANOVA). Tertiles of diet factor scores were used to examine the interaction and the main effects of club sports participation and dietary pattern on motor competence and body weight via 2 × 3 MANOVA (sports participation × diet factor tertiles). In a secondary multivariate analysis of covariance (MANCOVA), sex and BMI percentiles were included as covariates (2 × 3 MANCOVA).

## 3. Results

A total of 165 middle school students (55% male) provided complete data on food intake and motor competence. Descriptive data of the sample are shown in Table 1. Boys were significantly older than girls, and accordingly, were taller and heavier. There was, however, no sex difference in BMI percentile and the prevalence of overweight/obesity (girls: 17.1%, boys: 23.2%, *p* = 0.358). Almost half of the sample (48.5%) reported participation in club sports, with no difference in the sports participation rate between boys and girls (boys: 46.2%, girls: 51.4%, *p* = 0.506). Based on the absolute performance scores, boys performed significantly better than girls in the standing long jump and sit ups (*p* < 0.001) while girls performed better than boys in the stand and reach test (*p* < 0.001). Using age- and sex-normalized values, girls displayed higher scores compared to boys in the stand and reach test, sit ups, and 6-min run (Table 1). Boys, on the other hand, performed significantly better than girls at the 20 m sprint when using age- and sex-normalized values. Sex differences in total motor competence were borderline significant (*p* = 0.056), with girls having higher values than boys.

Dietary pattern also differed between boys and girls. Specifically, girls reported less frequent consumption of meat and soft drinks compared to boys, while their consumption of fruits and vegetables was more frequent (Table 2). This resulted in significantly lower scores on the meat/CHO factor (*p* = 0.020) in girls, while their score was higher for the water/low FF factor, compared to boys (*p* = 0.006). No significant sex difference occurred for the milk/cereal factor (*p* = 0.225).

Dietary patterns did not differ by sports participation. The association between sports participation and meat/CHO intake, however, reached borderline significance (*p* = 0.057), with lower values occurring in adolescents participating in club sports. There was no difference in the prevalence of overweight/obesity by club sports participation, and across tertiles of diet factors. Also, no interaction effects of diet pattern and club sports participation on BMI percentile were observed.

Motor competence, however, was significantly associated with club sports participation and dietary pattern. Combined associations of diet and sports participation with motor competence, however, were limited. The only significant combined association was observed between club sports participation and milk/cereal consumption on backwards balancing (*p* = 0.008), with a stronger association between dietary pattern and motor competence in adolescents not participating in club sports (Table 3).

**Figure 1:**
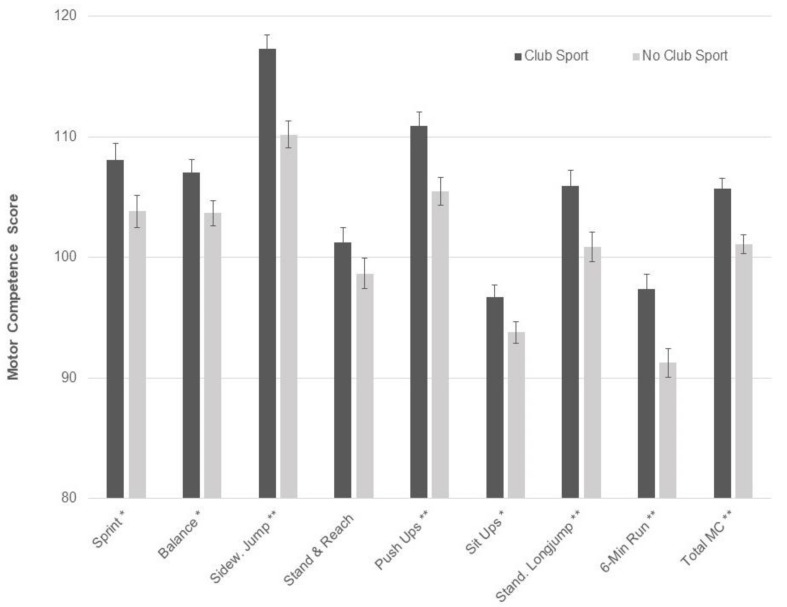
Main effects of club sports participation on motor competence based on 2 × 3 multivariate analysis of variance (MANOVA) (club sports by Water/low fast food (FF)). Values are sex-and age-normalized means with S.E.; * *p* < 0.050 ** *p* < 0.010. MC: motor competence.

**Figure 2:**
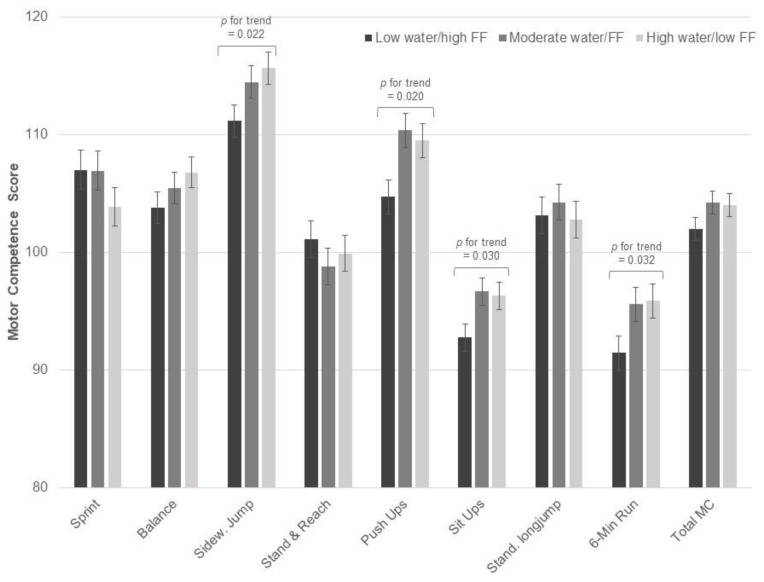
Main effects of club sports participation on motor competence based on 2 × 3 MANOVA (club sports by Water/low FF). Values are sex-and age-normalized means with S.E.

Several independent associations of club sports participation and dietary pattern on motor performance were observed. Specifically, club sports participation was associated with better performance on all individual motor competence test items (*p* < 0.050), except for the stand and reach test, resulting in better overall motor competence in participants reporting club sports, compared to those not reporting club sports (Figure 1). Regarding dietary patterns, higher water/low FF consumption was associated with better performance on sideways jumping (*p* for trend = 0.022), push-ups (*p* for trend = 0.020), sit ups (*p* for trend = 0.030), and 6-min run (*p* for trend = 0.032) (Figure 2). Further, lower scores on the milk/cereal factor were associated with better standing long jump performance and total motor competence (*p* for trend < 0.050). There was no significant association between meat/CHO consumption and motor competence. All previously reported results remained essentially unchanged after adjusting for sex and BMI percentile.

## 4. Discussion

Even though several studies have examined the association between sports/PA and motor competence [40,41,42,43], there exists limited research on the association between motor competence and dietary pattern. To the authors’ knowledge, this was also the first study that examined the combined association of dietary pattern and club sports participation with motor competence in Austrian adolescents. While there were no significant associations between dietary patterns and club sports participation, the present study showed independent associations of dietary pattern, as well as club sports participation with motor competence in middle school students. Specifically, club sports participation and healthier dietary choices (i.e., high water and low fast food/soft drink consumption) were associated with higher motor competence. High milk/cereal consumption, on the other hand, was associated with lower motor competence, particularly in participants not reporting club sports. These associations were independent of body weight, and neither club sports participation nor dietary pattern was associated with body weight in the present study.

The positive association between club sports participation and motor competence is consistent with previous research [43,44,45]. Longitudinal studies further indicate that the strength and directionality of the association between motor competence and club sports participation, as well as PA change over time [46,47]. Particularly during adolescence, motor competence appears to be an important facilitator and precursor for participation in sports clubs, while high PA and sports participation may be a prerequisite for motor development during childhood [45,46,47]. Accordingly, children and adolescents enter either a positive spiral of high PA, including sports participation and increased motor competence, or a vicious cycle of low motor competence and disengagement from sports. The importance of motor competence for sustainable participation in sports during adolescence may be attributed to an easier acquisition of sport-specific skills in children with higher motor competence. Higher motor competence also enhances self-efficacy, which facilitates participation in PA and sports [46]. In addition to actual motor competence, perceived competence appears to play an important role in the motivation for participation in sports [48], which is an important component to continuous engagement in various forms of PA.

Participation in organized sports has also been associated with other healthy lifestyle choices, including diet [29,49,50,51]. The present study, however, did not show healthier dietary patterns in club sports participants. Other studies also reported inconsistent results for the association between club sports participation and dietary pattern [29,52,53]. Even though sports participation has been associated with higher intake of fruits and vegetables, club sports participants also have been shown to consume high amounts of fast food and sugar-sweetened beverages [52]. In fact, it has been argued that sports participation during middle school is a strong risk factor for high fast food consumption during high-school years [54]. The higher fast food consumption in sports participants may be attributed to a more irregular eating pattern and a lower amount of meals consumed at home. The higher energy needs of more active adolescents may also contribute to the consumption of more energy dense foods, including fast foods. The results of the present study, nevertheless, indicate beneficial associations of a healthy dietary pattern with motor competence, independent of club sports participation. Specifically, healthier dietary choices were associated with better performance on agility, strength, and endurance tests. Previous studies also reported increased cardiorespiratory fitness with healthier dietary choices, particularly during adolescence [33,35,55]. While this may, at least partially, be attributed to an indirect association between body weight and motor competence [56], there was no difference in motor competence between overweight/obese and normal weight adolescents in the present study. Another possible explanation, therefore, could be that healthier dietary patterns indicate a greater parental support for a healthy lifestyle in general, including the facilitation of PA. Accordingly, parents may facilitate exposure to diverse movement experiences, which would facilitate motor development, even in the absence of club sports participation. Further, dietary pattern has been associated with sedentary choices, which also affect motor development [47]. Specifically, high media time has been associated with poorer diet quality [57,58] as well as low motor competence [47,59].

Sedentary behavior and total PA, rather than participation in club sports, are also crucial correlates of body weight [60]. Neither sports participation nor dietary pattern, however, was associated with body weight in the present study. Results on the association between club sports and body weight have generally been inconsistent [52], which may emphasize the importance of total PA rather than sports in weight management. In fact, it has been argued that a large amount of time in youth sport is spent sedentary or in only light PA [61,62]. Similarly, controversy remains on the relationship between diet and adiposity in youth [30]. Even though several studies showed an inverse association between dietary intake and body weight [63,64], there are also studies that did not show any association [65], or even direct associations between diet an body weight [66,67]. At least partially, this may be attributed to problems in obtaining accurate dietary data, particularly in youth [32]. It should, however, also be considered that more active youth have higher energy needs [31]. Accordingly, children and adolescents with high caloric intake may be able to maintain a healthy body weight as long as they are sufficiently active.

Several limitations of this study, however, need to be considered when interpreting the results. There was no objective measurement of total PA in the present study. Due to the reliance on self-reporting, club sports participation was used as an indicator for PA, as this may be reported more accurately than total PA. Previous research also indicated that participation in club sports is directly associated with total PA [52]. The sample distribution, however, did not allow for a differentiation by the amount of participation in club sports (e.g., hours, days); rather, only participants vs. non-participants could be analyzed. An additional limitation is that participants reported frequency rather than total amount of foods consumed, which provides only limited information on the total energy content of the diet. There is also an inherent risk of selective over- or under-reporting, due to social desirability and social approval with any form of diet report. Participants may have difficulties remembering all the foods, and some foods that they consumed may not have been listed on the questionnaire, which could have affected the reported dietary pattern. The cross-sectional nature of the study further does not allow for the establishment of causal relationships and temporal trends between sports participation, dietary pattern, and motor competence. In addition, the generalizability of the results may be limited, due to the small sample size and homogeneity of the study population. The objective assessment of various components of motor competence with a widely used and previously validated test, on the other hand, should be considered a strength of the study.

## 5. Conclusions

PA and healthy dietary habits play a crucial role in the development and general health of children and adolescents [1]. The present study also showed that both behaviors are independently associated with motor competence, which is an important component in the facilitation of an active lifestyle [36]. The facilitation of participation in sports, along with the promotion of healthy dietary choices may be particularly important during adolescence, as this is a critical time for the development of future lifestyle choices [3]. Accordingly, coaches, parents, and youth need to be educated on the importance of adequate nutrition in addition to participation in various forms of PA, including sports, for optimal motor development. Even though this may require additional efforts and resources, it may be a worthwhile investment to enhance the health and well-being of future generations.

## Figures and Tables

**Table 1:** Anthropometric characteristics and motor competence for the total sample and separately for girls and boys. Values are mean ± SD.

	Total Sample (*N* = 165)	Girls (*N* = 74)	Boys (*N* = 91)	*p* *
Age (years)	12.9 ± 1.2	12.6 ± 1.1	13.1 ± 1.2	0.009
Height (cm)	161.3 ± 8.9	158.8 ± 6.8	163.4 ± 9.9	0.001
Weight (kg)	53.8 ± 14.3	51.3 ± 9.9	55.9 ± 16.9	0.043
BMI percentile	59.4 ± 29.4	60.9 ± 27.5	58.2 ± 31.0	0.563
20 m sprint (sec)	3.8 ± 0.4	3.9 ± 0.4	3.7 ± 0.4	0.023
Balance (steps)	38.2 ± 9.1	39.5 ± 7.5	37.2 ± 10.2	0.755
Sideways jump (# in 15 s)	42.1 ± 7.5	41.4 ± 7.0	42.6 ± 7.9	0.554
Stand and reach (cm)	−0.3 ± 9.7	3.8 ± 8.5	−3.6 ± 9.3	0.011
Push-ups (# in 40 s)	15.6 ± 3.9	15.6 ± 3.4	15.6 ± 4.2	0.067
Sit ups (# in 40 s)	23.9 ± 4.6	22.4 ± 3.8	25.1 ± 4.9	0.011
Standing long jump (cm)	171.0 ± 29.7	159.5 ± 25.2	180.3 ± 29.8	0.648
6 min run (m)	997 ± 157	988 ± 119	1005 ± 183	<0.001
Total motor competence	103.3 ± 7.7	104.6 ± 6.9	102.3 ± 8.1	0.056

* *p*-value based on age- and sex-normalized values. # number of repetitions performed. BMI: Body mass index.

**Table 2:** Frequency of consumption of food groups (days/week). Values are Means ± SD.

	Total Sample	Girls	Boys	*p*
Meat	2.5 ± 1.2	2.1 ± 1.1	2.7 ± 1.2	0.002
Fish & Eggs	1.8 ± 1.2	1.6 ± 1.0	2.0 ± 1.3	0.051
Milk	2.2 ± 1.0	2.2 ± 1.1	2.3 ± 1.0	0.458
Carbs	2.2 ± 1.1	2.2 ± 1.0	2.2 ± 1.1	0.702
Bread	3.2 ± 0.9	3.3 ± 0.8	3.1 ± 1.0	0.371
Nuts & Seeds	1.2 ± 0.9	1.2 ± 1.0	1.2 ± 0.9	0.662
Fast Food	1.6 ± 0.9	1.5 ± 0.8	1.7 ± 0.9	0.105
Sweets	2.6 ± 1.1	2.5 ± 1.1	2.7 ± 1.2	0.420
Fruits	2.7 ± 1.2	2.9 ± 1.3	2.6 ± 1.1	0.044
Veggie	2.5 ± 1.5	2.9 ± 1.6	2.2 ± 1.4	0.003
Soft Drink	2.3 ± 1.3	2.0 ± 1.1	2.6 ± 1.3	0.001
Water	4.4 ± 1.6	4.6 ± 1.7	4.2 ± 1.5	0.126

**Table 3:** Motor competence by milk/veggie consumption and sports participation. Values are sex-and age-normalized Means ^†^ ± SD.

	Low Milk/Cereal		Moderate Milk/Cereal		High Milk/Cereal	
	Club Sports	No Club Sports	Club Sports	No Club Sports	Club Sports	No Club Sports
20 m sprint ^1^	108.9 ± 13.0	107.5 ± 9.5	106.9 ± 10.7	103.8 ± 14.1	108.1 ± 12.5	99.5 ± 13.0
Balance ^1,3,^*	104.6 ± 9.6	107.6 ± 8.2	107.8 ± 8.8	99.4 ± 10.2	108.4 ± 9.8	103.6 ± 11.1
Sideways jump ^1,^*	118.0 ± 6.9	111.6 ± 9.7	116.7 ± 7.9	111.7 ± 12.6	117.6 ± 7.9	110.1 ± 12.3
Stand and reach	101.5 ± 12.1	102.0 ± 9.3	101.9 ± 12.0	97.8 ± 12.3	100.3 ± 11.8	98.7 ± 11.1
Push-ups ^1,^*	111.2 ± 9.6	109.1 ± 7.9	110.1 ± 11.5	104.3 ± 13.3	111.5 ± 10.8	102.2 ± 10.9
Sit ups ^1^	97.3 ± 8.7	96.9 ± 7.7	96.0 ± 9.2	92.1 ± 10.4	97.0 ± 7.4	91.8 ± 7.5
Standing long jump ^1,^*^,2^	107.2 ± 9.2	104.2 ± 9.2	105.5 ± 12.6	100.6 ± 13.7	105.1 ± 11.0	97.4 ± 11.7
6 min run ^1,^*	97.0 ± 10.1	94.1 ± 11.4	98.3 ± 9.1	89.3 ± 12.2	97.0 ± 9.6	89.8 ± 11.6
Total motor competence^† 1,^*^,2^	105.8 ± 6.4	104.2 ± 5.8	105.5 ± 6.3	100.0 ± 9.4	105.8 ± 7.1	98.4 ± 7.5

^†^ Values >100 indicate above-average performance, while values <100 indicate below-average performance. ^1^ Significant main effect for club sport (*p* < 0.050; * *p* < 0.010). ^2^ Significant main effect for milk/veggie consumption (*p* for trend < 0.050; * *p* < 0.010). ^3^ Significant interaction effect of milk/veggie consumption and club sport participation (*p* < 0.050; * *p* < 0.010).

## References

[1] Office of the Surgeon General (US and National Institutes of Health) (2001). The Surgeon General’s Call to Action to Prevent and Decrease Overweight and Obesity.

[2] Resnick M.D., Catalano R.F., Sawyer S.M., Viner R., Patton G.C. (2012). Seizing the opportunities of adolescent health. Lancet.

[3] Sawyer S.M., Afifi R.A., Bearinger L.H., Blakemore S.J., Dick B., Ezeh A.C., Patton G.C. (2012). Adolescence: A foundation for future health. Lancet.

[4] US Department of Health and Human Services (2018). Physical Activity Guidelines for Americans.

[5] Institute of Medicine (2007). Nutrition Standards for Foods in Schools: Leading the Way Toward Healthier Youth.

[6] Kaiser-Jovy S., Scheu A., Greier K. (2017). Media use, sports activities, and motor fitness in childhood and adolescence. Wien. Klin. Wochenschr..

[7] Biddle S.J., Marshall S.J., Gorely T., Cameron N. (2009). Temporal and environmental patterns of sedentary and active behaviors during adolescents’ leisure time. Int. J. Behav. Med..

[8] Mathers M., Canterford L., Olds T., Hesketh K., Ridley K., Wake M. (2009). Electronic media use and adolescent health and well-being: Cross-sectional community study. Acad. Pediatr..

[9] Mensink G.B., Kleiser C., Richter A. (2007). [Food consumption of children and adolescents in Germany. Results of the German Health Interview and Examination Survey for Children and Adolescents (KiGGS)]. Bundesgesundheitsblatt Gesundheitsforschung Gesundheitsschutz.

[10] Krebs-Smith S.M., Guenther P.M., Subar A.F., Kirkpatrick S.I., Dodd K.W. (2010). Americans do not meet federal dietary recommendations. J. Nutr..

[11] Hardy L.L., Barnett L., Espinel P., Okely A.D. (2013). Thirteen-year trends in child and adolescent fundamental movement skills: 1997–2010. Med. Sci. Sports Exerc..

[12] Tomkinson G.R., Olds T.S. (2007). Secular changes in pediatric aerobic fitness test performance: The global picture. Med. Sport Sci..

[13] Lubans D.R., Morgan P.J., Cliff D.P., Barnett L.M., Okely A.D. (2010). Fundamental movement skills in children and adolescents: Review of associated health benefits. Sports Med..

[14] Holfelder B., Schott N. (2014). Relationship of fundamental movement skills in physical activity in children and adolescents: A systematic review. Psychol. Sport Exerc..

[15] Geidne S., Quennerstedt M., Eriksson C. (2013). The youth sports club as a health-promoting setting: An integrative review of research. Scand. J. Public Health.

[16] Badura P., Geckova A.M., Sigmundova D., van Dijk J.P., Reijneveld S.A. (2015). When children play, they feel better: Organized activity participation and health in adolescents. BMC Public Health.

[17] Hardy L.L., O’Hara B.J., Rogers K., St George A., Bauman A. (2014). Contribution of organized and nonorganized activity to children’s motor skills and fitness. J. Sch. Health.

[18] Hallal P.C., Wells J.C., Reichert F.F., Anselmi L., Victora C.G. (2006). Early determinants of physical activity in adolescence: Prospective birth cohort study. BMJ.

[19] Azevedo M.R., Araújo C.L., Cozzensa da Silva M., Hallal P.C. (2007). Tracking of physical activity from adolescence to adulthood: A population-based study. Rev. Saude Publica.

[20] Kokko S., Martin L., Geidne S., Van Hoye A., Lane A., Meganck J., Scheerder J., Seghers J., Villberg J., Kudlacek M. (2018). Does sports club participation contribute to physical activity among children and adolescents? A comparison across six European countries. Scand. J. Public Health.

[21] Hebert J.J., Møller N.C., Andersen L.B., Wedderkopp N. (2015). Organized Sport Participation Is Associated with Higher Levels of Overall Health-Related Physical Activity in Children (CHAMPS Study-DK). PLoS ONE.

[22] Mäkelä K., Kokko S., Kannas L., Villberg J., Vasankari T., Heinonen O., Savonen K., Alanko L., Korpelainen R., Selänne H. (2016). Physical activity, screen time, and sleep among youth participating and non-participating in organized sports—The Finnish health promoting Sports Club (FHPSC) Study. Adv. Phys. Educ..

[23] Marques A., Ekelund U., Sardinha L. (2016). Associations between organized sports participation and objectively measured physical activity, sedentary time and weight status in youth. J. Sci. Med. Sport.

[24] Hands B. (2008). Changes in motor skill and fitness measures among children with high and low motor competence: A five-year longitudinal study. J. Sci. Med. Sport.

[25] Ortega F.B., Ruiz J.R., Castillo M.J., Sjöström M. (2008). Physical fitness in childhood and adolescence: A powerful marker of health. Int. J. Obes..

[26] Eime R.M., Young J.A., Harvey J.T., Charity M.J., Payne W.R. (2013). A systematic review of the psychological and social benefits of participation in sport for adults: Informing development of a conceptual model of health through sport. Int. J. Behav. Nutr. Phys. Act..

[27] Badura P., Sigmund E., Geckova A.M., Sigmundova D., Sirucek J., van Dijk J.P., Reijneveld S.A. (2016). Is Participation in Organized Leisure-Time Activities Associated with School Performance in Adolescence?. PLoS ONE.

[28] Morris D. (2015). Actively closing the gap? Social class, organized activities, and academic achievement in high school. Youth Soc..

[29] Voráčová J., Badura P., Hamrik Z., Holubčíková J., Sigmund E. (2018). Unhealthy eating habits and participation in organized leisure-time activities in Czech adolescents. Eur. J. Pediatr..

[30] Moreno L.A., Rodríguez G. (2007). Dietary risk factors for development of childhood obesity. Curr. Opin. Clin. Nutr. Metab. Care.

[31] Lahoz-García N., García-Hermoso A., Milla-Tobarra M., Díez-Fernández A., Soriano-Cano A., Martínez-Vizcaíno V. (2018). Cardiorespiratory Fitness as a Mediator of the Influence of Diet on Obesity in Children. Nutrients.

[32] Cuenca-García M., Ortega F.B., Huybrechts I., Ruiz J.R., González-Gross M., Ottevaere C., Sjöström M., Dìaz L.E., Ciarapica D., Molnar D. (2012). Cardiorespiratory fitness and dietary intake in European adolescents: The Healthy Lifestyle in Europe by Nutrition in Adolescence study. Br. J. Nutr..

[33] Zaqout M., Vyncke K., Moreno L.A., De Miguel-Etayo P., Lauria F., Molnar D., Lissner L., Hunsberger M., Veidebaum T., Tornaritis M. (2016). Determinant factors of physical fitness in European children. Int. J. Public Health.

[34] Howe A.S., Skidmore P.M., Parnell W.R., Wong J.E., Lubransky A.C., Black K.E. (2016). Cardiorespiratory fitness is positively associated with a healthy dietary pattern in New Zealand adolescents. Public Health Nutr..

[35] Saeedi P., Black K.E., Haszard J.J., Skeaff S., Stoner L., Davidson B., Harrex H.A.L., Meredith-Jones K., Quigg R., Wong J.E. (2018). Dietary Patterns, Cardiorespiratory and Muscular Fitness in 9⁻11-Year-Old Children from Dunedin, New Zealand. Nutrients.

[36] Drenowatz C. (2017). A focus on motor competence as alternative strategy for weight management. J. Obes. Chron. Dis..

[37] Kromeyer-Hauschild K., Wabitsch M., Kunze D., Geller F., Geiß H., Hesse V., von Hippel A., Jaeger U., Johnson D., Korte W. (2001). Perzentile für den Body-mass-Index für das Kindes- und Jugendalter unter Heranziehung verschiedener deutscher Stichproben. Monatsschrift Kinderheilkunde.

[38] Bös K., Schlenker L., Büsch D., Lämmle L., Müller H., Oberger J., Seidl I., Tittlbach S. (2009). Deutscher Motorik-Test 6-18 (DMT6-18) [German Motor Abilities Test 6-18 (DMT6-18)].

[39] Greier K., Ruedl G., Weber C., Thöni G., Riechelmann H. (2016). Ernährungsverhalten und motorische Leistungsfähigkeit von 10- bis 14-jährigen Jugendlichen. Ernährung Medizin.

[40] D’Hondt E., Deforche B., Gentier I., De Bourdeaudhuij I., Vaeyens R., Philippaerts R., Lenoir M. (2013). A longitudinal analysis of gross motor coordination in overweight and obese children versus normal-weight peers. Int. J. Obes..

[41] Vandorpe B., Vandendriessche J., Vaeyens R., Pion J., Matthys S., Lefevre J., Philippaerts R., Lenoir M. (2012). Relationship between sports participation and the level of motor coordination in childhood: A longitudinal approach. J. Sci. Med. Sport.

[42] Okely A.D., Booth M.L., Patterson J.W. (2001). Relationship of physical activity to fundamental movement skills among adolescents. Med. Sci. Sports Exerc..

[43] Jaakkola T., Kalaja S., Liukkonen J., Jutila A., Virtanen P., Watt A. (2009). Relations among physical activity patterns, lifestyle activities, and fundamental movement skills for Finnish students in grade 7. Percept. Mot. Skills.

[44] Campos C., Queiroz D., Silva J., Feitoza A., Cattuzzo M. (2017). Relationship between organized physical activity and motor competence in teenagers. Am. J. Sport Sci. Med..

[45] Fransen J., Deprez D., Pion J., Tallir I.B., D’Hondt E., Vaeyens R., Lenoir M., Philippaerts R.M. (2014). Changes in physical fitness and sports participation among children with different levels of motor competence: A 2-year longitudinal study. Pediatr. Exerc. Sci..

[46] Stodden D., Goodway J., Langendorfer S., Roberton M., Rudisill M., Garcia C., Garcia L. (2008). A developmental perspective on the role of motor skill competence in physical activity: An emergent relationshihp. Quest.

[47] Drenowatz C., Greier K. (2019). Cross-sectional and longitudinal assocaition between club sports participation, media consumption and motor competence in adolescents. Scand. J. Med. Sci. Sports.

[48] Khodaverdi Z., Bahram A., Stodden D., Kazemnejad A. (2016). The relationship between actual motor competence and physical activity in children: Mediating roles of perceived motor competence and health-related physical fitness. J. Sports Sci..

[49] Torstveit M.K., Johansen B.T., Haugland S.H., Stea T.H. (2018). Participation in organized sports is associated with decreased likelihood of unhealthy lifestyle habits in adolescents. Scand. J. Med. Sci. Sports.

[50] Taliaferro L.A., Rienzo B.A., Donovan K.A. (2010). Relationships between youth sport participation and selected health risk behaviors from 1999 to 2007. J. Sch. Health.

[51] Dortch K.S., Gay J., Springer A., Kohl H.W., Sharma S., Saxton D., Wilson K., Hoelscher D. (2014). The association between sport participation and dietary behaviors among fourth graders in the school physical activity and nutrition survey, 2009–2010. Am. J. Health Promot..

[52] Nelson T.F., Stovitz S.D., Thomas M., LaVoi N.M., Bauer K.W., Neumark-Sztainer D. (2011). Do youth sports prevent pediatric obesity? A systematic review and commentary. Curr. Sports Med. Rep..

[53] Vella S.A., Cliff D.P., Okely A.D., Scully M.L., Morley B.C. (2013). Associations between sports participation, adiposity and obesity-related health behaviors in Australian adolescents. Int. J. Behav. Nutr. Phys. Act..

[54] Bauer K.W., Larson N.I., Nelson M.C., Story M., Neumark-Sztainer D. (2009). Socio-environmental, personal and behavioural predictors of fast-food intake among adolescents. Public Health Nutr..

[55] Arriscado D., Muros J.J., Zabala M., Dalmau J.M. (2014). Factors associated with low adherence to a Mediterranean diet in healthy children in northern Spain. Appetite.

[56] Greier K., Drenowatz C. (2018). Bidirectional association between weight status and motor skills in adolescents: A 4-year longitudinal study. Wien. Klin. Wochenschr..

[57] Kremers S.P., van der Horst K., Brug J. (2007). Adolescent screen-viewing behaviour is associated with consumption of sugar-sweetened beverages: The role of habit strength and perceived parental norms. Appetite.

[58] Utter J., Scragg R., Schaaf D. (2006). Associations between television viewing and consumption of commonly advertised foods among New Zealand children and young adolescents. Public Health Nutr..

[59] Mota J., Ribeiro J.C., Carvalho J., Santos M.P., Martins J. (2010). Television viewing and changes in body mass index and cardiorespiratory fitness over a two-year period in schoolchildren. Pediatr. Exerc. Sci..

[60] Must A., Tybor D.J. (2005). Physical activity and sedentary behavior: A review of longitudinal studies of weight and adiposity in youth. Int. J. Obes..

[61] Leek D., Carlson J.A., Cain K.L., Henrichon S., Rosenberg D., Patrick K., Sallis J.F. (2011). Physical activity during youth sports practices. Arch. Pediatr. Adolesc. Med..

[62] Wickel E.E., Eisenmann J.C. (2007). Contribution of youth sport to total daily physical activity among 6- to 12-yr-old boys. Med. Sci. Sports Exerc..

[63] Stallmann-Jorgensen I.S., Gutin B., Hatfield-Laube J.L., Humphries M.C., Johnson M.H., Barbeau P. (2007). General and visceral adiposity in black and white adolescents and their relation with reported physical activity and diet. Int. J. Obes..

[64] Telford R.D., Cunningham R.B., Telford R.M., Riley M., Abhayaratna W.P. (2012). Determinants of childhood adiposity: Evidence from the Australian LOOK study. PLoS ONE.

[65] McGloin A.F., Livingstone M.B., Greene L.C., Webb S.E., Gibson J.M., Jebb S.A., Cole T.J., Coward W.A., Wright A., Prentice A.M. (2002). Energy and fat intake in obese and lean children at varying risk of obesity. Int. J. Obes. Relat. Metab. Disord..

[66] Elliott S.A., Truby H., Lee A., Harper C., Abbott R.A., Davies P.S. (2011). Associations of body mass index and waist circumference with: Energy intake and percentage energy from macronutrients, in a cohort of Australian children. Nutr. J..

[67] Skinner A.C., Steiner M.J., Perrin E.M. (2012). Self-reported energy intake by age in overweight and healthy-weight children in NHANES, 2001–2008. Pediatrics.

